# Identifying perinatal risk factors for infant maltreatment: an ecological approach

**DOI:** 10.1186/1476-072X-5-53

**Published:** 2006-12-04

**Authors:** Yueqin Zhou, Elaine J Hallisey, Gordon R Freymann

**Affiliations:** 1 Department of Geosciences, Georgia State University, 340 Kell Hall, 24 Peachtree Center Ave., P.O. Box 4105, Atlanta, GA 30303, USA; 2 Office of Health Information & Policy, Division of Public Health, Georgia Department of Human Resources, 2 Peachtree Street, Atlanta, GA 30303, USA

## Abstract

**Background:**

Child maltreatment and its consequences are a persistent problem throughout the world. Public health workers, human services officials, and others are interested in new and efficient ways to determine which geographic areas to target for intervention programs and resources. To improve assessment efforts, selected perinatal factors were examined, both individually and in various combinations, to determine if they are associated with increased risk of infant maltreatment. State of Georgia birth records and abuse and neglect data were analyzed using an area-based, ecological approach with the census tract as a surrogate for the community. Cartographic visualization suggested some correlation exists between risk factors and child maltreatment, so bivariate and multivariate regression were performed. The presence of spatial autocorrelation precluded the use of traditional ordinary least squares regression, therefore a spatial regression model coupled with maximum likelihood estimation was employed.

**Results:**

Results indicate that all individual factors or their combinations are significantly associated with increased risk of infant maltreatment. The set of perinatal risk factors that best predicts infant maltreatment rates are: mother smoked during pregnancy, families with three or more siblings, maternal age less than 20 years, births to unmarried mothers, Medicaid beneficiaries, and inadequate prenatal care.

**Conclusion:**

This model enables public health to take a proactive stance, to reasonably predict areas where poor outcomes are likely to occur, and to therefore more efficiently allocate resources. U.S. states that routinely collect the variables the National Center for Health Statistics (NCHS) defines for birth certificates can easily identify areas that are at high risk for infant maltreatment. The authors recommend that agencies charged with reducing child maltreatment target communities that demonstrate the perinatal risks identified in this study.

## Background

Child maltreatment, including neglect, physical abuse, emotional abuse, sexual abuse, and other types of abuse, is a persistent problem in the world, not only in poor countries, but also in rich, industrialized nations, including the United States [1]. In general, the rate of child maltreatment is inversely related to the age of the child: children from 0 to 3 years of age have the highest rate [2]. Infants, children under age one, have the highest percentage of fatalities. Infants also suffer more serious physical and developmental consequences from maltreatment [3].

In addition to immediate effects, child maltreatment has pronounced long-term negative medical and social consequences. Numerous studies in medical literature confirm the association between childhood maltreatment and adverse adult health outcomes [4]. Examples include smoking [5], drug abuse [6], depression [7,8], stress disorders [9], and certain chronic diseases [10]. For example, a study on the continuing consequences of maltreatment in the early years using longitudinal data from infancy through late adolescence confirmed the adverse impact of early maltreatment on later antisocial behavior [11].

In addition to medical and social consequences, the economic impact of child maltreatment is immense. Nationwide costs resulting from abuse and neglect are estimated to be as high as $94 billion per year, of which $24.3 billion are used for the immediate needs of abused or neglected children including hospitalization, treatment of chronic health problems, mental health care, child welfare, law enforcement, and the judicial system; the remaining $69.7 billion are costs associated with long-term and/or secondary effects of child abuse and neglect [12]. A study assessing the economic burden of hospitalization associated with child abuse and neglect found that children whose hospitalization was due to abuse or neglect were significantly more likely to have longer hospital stays, with double the total charges of other hospitalized children, and that nearly two-thirds of the primary payer were Medicaid [13].

A recent study, focusing on infant maltreatment assessment, found that a set of perinatal risk factors significantly influences the probability that an infant would be maltreated by caregivers [14]. In this cohort study, the researchers studied 15 perinatal risk factors using data of 1,602 infant victims of maltreatment among 189,055 infants born in 1996 in Florida. They found 11 factors were significantly related to infant maltreatment, five of which had adjusted relative risks of two or greater. The five key factors are: mother smoked during pregnancy, more than two siblings, Medicaid beneficiary, unmarried marital status, and birth weight less than 2,500 grams. Infants who had four of these five risk factors had a maltreatment rate seven times higher than the population average. Other significant risk factors include maternal age less than 20, maternal education less than high school, and prenatal care, as measured by the Kotelchuck Index, less than adequate.

Another study linked premature births to substantiated infant abuse [15]. The researchers concluded that the cry of the premature infants was perceived to be more aversive than the cry of full-term infants, and elicited greater arousal and subsequent abuse. Bugental and Happaney also identified low 5-minute Apgar scores and premature births as predictor variables of infant maltreatment [16].

Given the negative consequences associated with infant maltreatment, in conjunction with the need to efficiently allocate resources, the goal of our study was to develop a population-based model that enables public health agencies to identify areas at high risk for infant maltreatment. Many studies fail to consider an ecological, population-based method that explores why these individual risk factors occur in the larger context of the environment in which they are found. The aforementioned risk factors are themselves outcomes and an ecological approach may help better demonstrate and understand the presence of underlying social and spatial processes that predispose certain caregivers to maltreat. The Health Field Concept [17] and the Health Field Theory [18] consider the interactions of biology, environment, lifestyle, and health system effects and capacities as the major determinants of health. Both take a holistic view of community assessment that supports the ecological approach.

To achieve this goal, we examined the geographic distribution of infant maltreatment in relation to relevant perinatal risk factors, as identified in previous research, using Georgia data aggregated by census tract. Two major components of the goal were: 1) to determine if infant maltreatment, including all types of abuse and neglect combined, is significantly related to a set of individual and composite perinatal risk factors, and 2) to identify a set of risk factors that best predicts infant maltreatment rates to ultimately aid public health agencies in identifying geographic areas for intervention.

Special attention was focused on coping with spatial autocorrelation, a well-known spatial phenomenon in which data collected from a particular location is often similar to data collected in nearby locations. The presence of spatial autocorrelation violates the key assumption of independence for regression analyses. Researchers working with spatially aggregated data have noted that when spatial autocorrelation exists in the data but is ignored in analyses, such as in classic ordinary least squares (OLS) regression, the results are biased. To derive reliable results, spatial analytical techniques were used to control for the effects of spatial autocorrelation [19].

## Methods

### Data

Two sources of data were used in this study: substantiated neglect and abuse data from the Division of Family and Children Services (DFCS), and vital record births from the Division of Public Health (DPH), both of the Georgia Department of Human Resources (DHR). The State of Georgia complies with the U.S. Standard Certificate of Live Birth, developed by the National Center for Health Statistics (NCHS) [20], with regards to the variables acquired for birth certificates. This research was undertaken with the approval of the DHR Institutional Review Board (IRB). Given that the research involved the use of existing data sets, with confidentiality preserved, there were no risks to individual human subjects. The DHR Project Number is 060807.

Data on substantiated neglect and abuse were collected from 2000 through 2002 between January 1^st ^and December 31^st ^of each year. In 2000, 2001, and 2002, respectively, 2,642, 2,869, and 3,205 infants were victims of one or more types of maltreatment including neglect, physical abuse, emotional abuse, sexual abuse, and other types of abuse. DFCS declined to provide identifiers to link substantiated maltreatment cases to births, so we were unable to conduct the study at the individual level; instead, we conducted an ecological study. That is, we examined child maltreatment in relation to perinatal characteristics of the communities in which the maltreatment victims lived [21]. The census tract is a surrogate for the community. Tracts are sub-county areal units designed to be demographically homogeneous by the U.S. Bureau of the Census. The outcome variable is the rate of infant maltreatment by census tract. The hypothesis is that a census tract having a higher percentage of births with perinatal risks is more likely to have higher rate of infant maltreatment.

We followed the Standard Geocoding Procedures, designed by and applied within the Office of Health Information and Policy (OHIP), DPH, Georgia DHR, to assign latitude and longitude values to each of the records (F. Millard and G. Freymann, unpublished work, December 2001). The individual records were then aggregated to determine the number of maltreatment events (all types combined) in each census tract.

We extracted the birth records for 1999 through 2002, from which we derived risk factors and calculated the counts of births.

Individual perinatal risk factors examined include:

1. Medicaid beneficiary

2. Unmarried mother

3. Maternal age less than 20 years of age

4. Maternal education less than high school

5. Three or more siblings

6. Prenatal care less than adequate

7. Birth weight less than 2,500 grams

8. Mother smoked during pregnancy

9. Gestation less than 37 weeks

10. 5-minute Apgar scores less than 7

Composite risks include:

11. Child_risk: Any of risks 7, 9, or 10 (representing neonatal difficulties) is present.

12. Composite_risk1: Of risks 1, 2, 5, 7, or 8, three or more are present.

13. Composite_risk2: Of risks 1, 2, 5, 8, or 11, three or more are present.

The child risk composite (Child_risk) is a single variable that represents the presence of one or more neonatal difficulties. Composite_risk1 characterizes extreme risk infants who had three or more key risk factors present (identified in [14]). Composite_risk2 is equivalent to Composite_risk1 except the child risk composite replaces low birth weight.

To adjust for the effects of different lengths of exposure in each year, we applied the person-time concept to calculate the rate of infant maltreatment for each tract by dividing the total number of maltreatment victims by the total number of weighted births during a three-year period [22]. Births were weighted by the length of time spent, as an infant, in each target calendar year of data collection. In doing so, we calculated for each record three weighting fields, each denoting the proportion of time, in infancy, over a one-year period. The number of weighted births for a census tract was the sum of all three weights of all the births residing in that tract. Then the numbers of maltreatment victims and weighted births for individual years for each tract were summed to obtain the total number of maltreatment victims and total number of weighted births during a three-year period.

**Figure 1:**
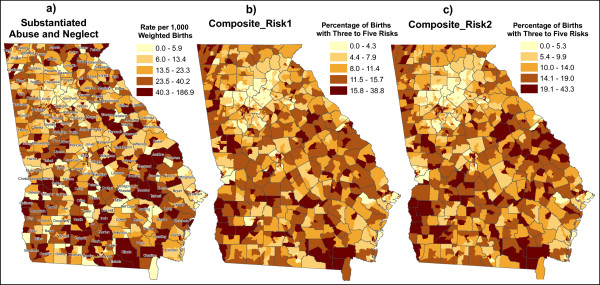
**Cartographic visualization of infant maltreatment in the state of Georgia**. **a)** Substantiated Abuse and Neglect. Birth is weighted by the length of time as an infant in the target year. **b)** Composite_Risk1. Of the 10 perinatal risks examined, this composite includes: 1. Medicaid beneficiary; 2. Unmarried mother; 5. Infant with three or more siblings; 7. Birth weight < 2500 g; 8. Mother smoked during pregnancy. **c)** Composite_Risk2. Of the 10 perinatal risks examined, this composite includes: 1. Medicaid beneficiary; 2. Unmarried mother; 5. Infant with three or more siblings; 8. Mother smoked during pregnancy; 7., 9., or 10. Presence of neonatal difficulties.

Among the 1,618 census tracts in Georgia for the year 2000, 32 tracts had fewer than 30 weighted births. To reduce small number problems in calculating rates, each of these 32 tracts was merged to one or more neighboring tracts, resulting in 1,589 enumeration units (for simplicity still referred to as tracts), each with more than 30 weighted births. Figure 1a displays the substantiated infant maltreatment rates by tract, presented as the number of substantiated infant maltreatment victims per 1,000 weighted births.

All birth records for the years 1999 through 2002 were processed to create Boolean fields, 1 meaning present and 0 not present, for each risk factor. The Child_risk factor was coded 1 if any of the three previously-mentioned neonatal difficulties were present. Composite_risk1 or Composite_risk2 were coded 1 if three or more of the related 5 risk factors were present. For any record, if any of the factors were unknown, the record was omitted for the calculations of risk factors. The individual birth records were then aggregated to the tract, from which we obtained the percentage of births coded 1 in each tract for each of the risk factors. Figures 1b and 1c display the percentage of births coded 1 by tract for two composite risk factors (Composite_risk1 and Composite_risk2).

### Analysis

In any spatial analysis, reviewing mapped data is recommended to determine if the distribution suggests any patterns or relationships among mapped features [23]. Qualitative, visual analysis of the mapped data, or cartographic visualization (Figures 1a, 1b, and 1c), suggests that spatial autocorrelation, meaning that similar data values tend to cluster geographically, is more pronounced with regards to the risk factors (Figures 1b and 1c) in contrast to substantiated maltreatment (Figure 1a). In Figure 1a, tracts in the lowest classification are more often immediately adjacent to tracts in the highest classification (note that all maps use the quantiles classification method). For instance, tracts in Montgomery and Wheeler, adjacent counties in the southeastern portion of the state, are at opposite ends of the classification scheme in terms of maltreatment, whereas the risk factor maps show both Montgomery and Wheeler in the mid- to upper mid-range of the classification scheme.

**Figure 2:**
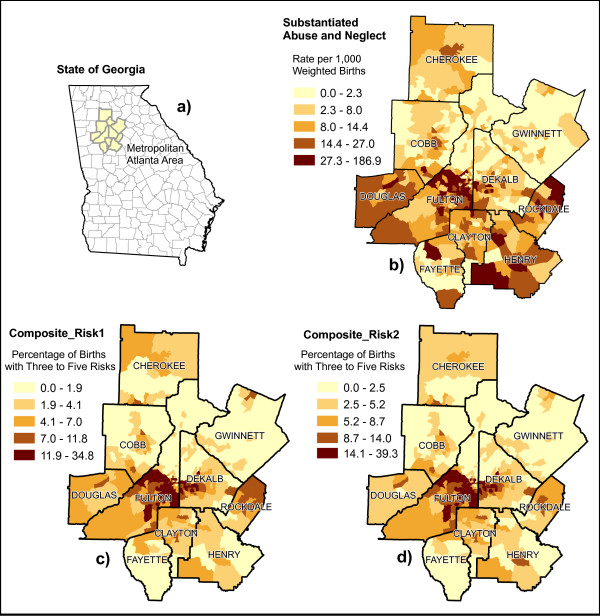
**Cartographic visualization of infant maltreatment in metropolitan Atlanta**. a) Inset map showing the location of the Atlanta area within the state of Georgia. b) Substantiated Abuse and Neglect. c) Composite_Risk1. d) Composite_Risk2.

Any correlation present among the three maps seems to be more readily visible in the urban areas of the state, as opposed to rural portions of the state. The north central counties of Fulton, DeKalb, Cobb, Gwinnett, and Clayton, which make up much of metropolitan Atlanta, show a clear pattern (Figure 2b). North Fulton County, which has a low rate of substantiated maltreatment, is in stark contrast to central and south Fulton, which have a high rate of substantiated maltreatment. Northern DeKalb County, immediately adjacent to Fulton, is also lower in risk than southern DeKalb. Additionally, the north metro counties of Gwinnett, and to a lesser extent Cobb, have low rates of substantiated maltreatment, in contrast to the south metro county of Clayton which has a higher rate of maltreatment. The pattern of substantiated maltreatment in Atlanta is echoed in both the maps of perinatal risk factors, with fewer risk factors in the north and more risk factors in the south (Figures 2c and 2d). Other urban areas in the state depict similar patterns among the three maps.

This visual analysis noting the presence of spatial autocorrelation and the correlation between infant maltreatment and risk factors is tenuous at best and must be validated with robust quantitative spatial analysis. The following paragraphs describe the quantitative spatial analysis of perinatal risk factors for substantiated infant maltreatment.

We used bivariate and multivariate linear regression methods for the quantitative analysis. The dependent variable was the maltreatment rate, and the predictor variables were the individual and composite risk factors. To ensure the normal distribution of the dependent variable, we transformed the rate to its natural logarithmic form using the following formula [24]:

zi=ln⁡(1000∗(Yi+1)WNi)     (1)
 MathType@MTEF@5@5@+=feaafiart1ev1aaatCvAUfKttLearuWrP9MDH5MBPbIqV92AaeXatLxBI9gBaebbnrfifHhDYfgasaacH8akY=wiFfYdH8Gipec8Eeeu0xXdbba9frFj0=OqFfea0dXdd9vqai=hGuQ8kuc9pgc9s8qqaq=dirpe0xb9q8qiLsFr0=vr0=vr0dc8meaabaqaciaacaGaaeqabaqabeGadaaakeaacqWG6bGEdaWgaaWcbaGaemyAaKgabeaakiabg2da9iGbcYgaSjabc6gaUjabcIcaOmaalaaabaGaeGymaeJaeGimaaJaeGimaaJaeGimaaJaey4fIOIaeiikaGIaemywaK1aaSbaaSqaaiabdMgaPbqabaGccqGHRaWkcqaIXaqmcqGGPaqkaeaacqWGxbWvcqWGobGtdaWgaaWcbaGaemyAaKgabeaaaaGccqGGPaqkcaWLjaGaaCzcamaabmaabaGaeGymaedacaGLOaGaayzkaaaaaa@47EB@

where ln() is the natural logarithmic transformation function;

*Y*_*i *_is the number of infant maltreatments in tract *i*;

*WN*_*i *_is the weighted births in tract *i*;

and *z*_*i *_is the transformed rate.

This formula not only gives valid values for those tracts with *Y*_*i *_= 0, but also helps discriminate the tracts with *Y*_*i *_≤ 1 but with different *WN*_*i *_[24].

Let *x*_1_, *x*_2_, ... *x*_*k *_denote *k *risk factors chosen to be included in the regression equation; *z *the dependent variable; and *e *the error term. A multivariate regression equation is expressed as:

*z *= *b*_0 _+ *b*_1_*x*_1 _+ *b*_2_*x*_2 _+ ... + *b*_*k*_*x*_*k *_+ *e *= *X*β + *e *    (2)

in which *X *= (1 *x*_1 _*x*_2 _... *x*_*k*_), β = [*b*_0 _*b*_1 _... *b*_*k*_]^T^, *b*_0 _is the constant, and *b*_*j*_(*j *= 1, ..., *k*) is the slope reflecting the relationship between *x*_*j *_and *z*. Equation (2) becomes the bivariate regression expression if only retaining a single risk variable.

In traditional statistics with nonspatial data, the OLS method is used to estimate parameters *b*_0_, *b*_1_, ..., *b*_*k *_(or *b*_0_, *b*_1 _in bivariate regression). In order for the statistical inference about parameter estimates to be valid, some assumptions about the data must be satisfied [25]. The key assumption is that the error term is independent and normally distributed with a constant mean of zero and constant variance of σ^2 ^(homogeneity). For multivariate regression, an additional key assumption is that there is no multicollinearity among the predictor variables.

Assuming there is no serious problem with multicollinearity, the OLS method provides the best linear unbiased estimates only if the regression model is correctly specified so that the error term meets the above assumption. A regression model is considered misspecified in several situations: 1) the dependent variable is inherently spatially dependent; 2) the unit of analysis does not match the unit of actual phenomena; 3) important risk variables are not included in the model; and 4) the observations of the dependent and/or predictor variables are not free of errors [24,26,27]. If a regression model is misspecified, the errors after the OLS fitting will not be independent; instead, the error at one location may be correlated with the errors at nearby locations, resulting in the clustering of similar errors among nearby locations, or spatial autocorrelation. When the errors are spatially autocorrelated, the OLS estimates are no longer unbiased and the goodness-of-fit measure *R*^2 ^is upward biased [28].

A common method to handle spatial autocorrelation is to minimize its effects by resampling to create a subset of data. This can be done in one of two ways; either manually selecting data locations or using a random process [29]. However, both the manual and the random process have some drawbacks. Manual selection may be subjective, random selection may not be free of spatial dependency, and both methods may result in loss of information, that is, the selected subset may not represent all the characteristics of the dataset.

A less commonly used but more objective method is spatial regression, which considers spatial autocorrelation an additional variable in the regression equation and solves its effect simultaneously with the effects of other explanatory variables [27]. This method uses all available information in the dataset.

We therefore controlled for spatial autocorrelation effects using the spatial regression method. There are two ways to incorporate spatial autocorrelation in a regression model. One is to model spatial autocorrelation in the error term as a spatially lagged dependent variable, and the other as a spatially lagged error term, that is,

*e *= ρ*Wz *+ ε     (3)

or

*e *= λ*We *+ ε     (4)

where *W *is the weight matrix characterizing the spatial relationship between every pair of observations;

ρ or λ is the spatial autoregressive parameter characterizing spatial autocorrelation;

And ε is the independent and normally distributed error term with a constant mean of zero and constant variance.

The former is referred to as a spatial lag model and the latter a spatial error model. Substituting the error term in (2) with Equation (3) or (4) and reorganizing the equation lead to the expression of a spatial lag model:

*z *= (*I *- ρ*W*)^-1^*X*β + (*I *- ρ*W*)^-1^* ε *    (5)

or that of a spatial error model

*z *= *X*β + (*I *- λ*W*)^-1^* ε *    (6)

where *I *is the identity matrix.

The OLS method is no longer appropriate for estimating the parameters in Equations (5) and (6); instead, the maximum likelihood estimation method (MLE) or the instrumental variables estimation (IVE) method should be used [26]. The MLE method estimates model parameters by maximizing the *Likelihood Function *of the observations [26,27].

When the MLE method is used to estimate the parameters in Equation (5) or (6), the traditional goodness-of-fit measure, *R*^2^, is no longer valid for assessing model fit [26]. One appropriate measure is the Akaike Information Criteria (AIC). A model is considered the best among a set of alternatives if the model gives the smallest AIC value. An approximate measure that mimics *R*^2 ^is the so-called pseudo-*R*^2^, which provides a measure of linear association between the observed and predicted values of the dependent variable, but is no longer related to the variance component explained by the model.

The software used for the spatial regression analysis in the present study is GeoDA (Version 0.9.5i_6) [30,31]. The program provides tools to calculate spatial weights, and run OLS (Classic) as well as spatial regression (Spatial Lag and Spatial Error) models. The output of the OLS models includes the diagnostic for multicollinearity (i.e., multicollinearity condition number or MCN), and the array of test statistics for spatial autocorrelation, which suggest whether spatial autocorrelation is significant to consider, and if so, which spatial model should be used. A value over 30 for MCN suggests problems with multicollinearity [32].

## Results

Table 1 lists the descriptive statistics of the dependent variable and risk factors. The rate is highly positively skewed (Skewness = 1.58) followed by three risk factors (Three or more siblings, 5-Minute Apgar Scores <7, and Mother smoked during pregnancy). As discussed previously, the transformation of rates reduced skewness. The transformed rates are slightly negatively skewed (Skewness = -0.37) with 7 lower outliers and no upper outliers. The lower outliers were excluded from the regression analyses.

**Table 1:** Descriptive statistics of infant maltreatment rates and perinatal risk factors

**Variables**	**Min**	**Max**	**Mean**	**Standard Deviation**	**Skewness**	**Number of outliers**	
						
						**Lower**	**Upper**
Infant maltreatment rates							

Rate	0.0	186.9	24.4	23.0	**1.58**	0	62
Transformed rate	0.4921	5.2844	3.1190	0.8225	-0.37	7	0

Perinatal risk factors: individual							

Gestation weeks <37	2.4	32.7	11.7	3.4	0.69	2	24
Birth weight <2500 g	0.0	24.6	8.0	3.2	0.78	0	22
5-Minute APGAR Score <7	0.0	7.8	1.3	1.0	**1.27**	0	46
Prenatal care less than adequate	0.4	57.1	23.4	11.4	0.40	0	0
Maternal age <20	0.0	39.3	14.9	7.6	0.10	0	3
Maternal education < HS	0.0	80.3	25.0	14.0	0.40	0	8
Unmarried mother	1.3	97.1	40.3	21.7	0.50	0	0
Three or more siblings	0.0	32.2	9.4	4.4	**1.32**	0	62
Mother smoked during pregnancy	0.0	46.2	9.4	6.6	**1.15**	0	31
Medicaid beneficiary	0.7	94.8	48.7	21.1	-0.34	0	0

Perinatal risk factors: composite							

Child_risk	2.4	34.7	15.0	4.3	0.55	1	15
Composite_risk1	0.0	38.8	10.5	6.9	0.79	0	29
Composite_risk2	0.0	43.3	12.8	8.1	0.70	0	23

The data were first analyzed using the OLS model. The errors, i.e., the differences between the observed and predicted values of the dependent variable, were computed and tested for the statistical significance of spatial autocorrelation. The test statistics (not shown) indicated that spatial autocorrelation was significant for all the bivariate and multivariate models. Therefore, spatial regression was performed to account for spatial autocorrelation effects. The results are presented in Tables 2 and 3. Table 2 presents the bivariate spatial regression results, in which Models 1–10 are related to individual risk factors, and Models 11–13 to composite risk factors. The smaller the AIC value, the better the model. Model 13, Composite_risk2, is the best among the bivariate models in terms of providing the smallest AIC value. All the individual risk factors as well as the three composite risk factors are statistically significantly with respect to the rate of infant maltreatment, each with probability *P*-value < 0.0000. It is noted that in each of the bivariate regression models spatial autocorrelation is statistically significant with *P*-value < 0.0000.

**Table 2:** Spatial regression of infant maltreatment rates on perinatal risk factors: bivariate

**Models**	**Variables**	**Coefficient Estimates**		***P*-value**	**Spatial auto-correlation estimates**	**AIC**	**Pseudo-*R*^2^**
						
		**Estimates**	**Standard Error (SE)**				
1	Medicaid beneficiary	0.0213	0.0009	0.0000	0.7133*	2648.5	0.541
2	Unmarried mother	0.0195	0.0009	0.0000	0.7840*	2683.6	0.537
3	Maternal age <20	0.0440	0.0024	0.0000	0.5653*	2701.2	0.518
4	Maternal education < HS	0.0246	0.0014	0.0000	0.7896*	2774.1	0.510
5	Three or more siblings	0.0558	0.0041	0.0000	0.8243*	2887.4	0.477
6	Prenatal care less than adequate	0.0294	0.0023	0.0000	0.8400*	2907.0	0.473
7	Birth weight <2500 g	0.0522	0.0051	0.0000	0.7827*	2950.4	0.452
8	Mother smoked during pregnancy	0.0327	0.0037	0.0000	0.8345*	2981.6	0.446
9	Gestation weeks <37	0.0393	0.0046	0.0000	0.8070*	2986.3	0.442
10	5-Minute APGAR Score < 7	0.0728	0.0152	0.0000	0.8388*	3036.6	0.428

11	Child_risk	0.0408	0.0038	0.0000	0.7733*	2941.9	0.454
12	Composite_risk1	0.0618	0.0028	0.0000	0.7281*	2649.7	0.542
**13**	Composite_risk2	0.0545	0.0024	0.0000	0.7219*	**2626.3**	0.548

*P-value < 0.0000

**Table 3:** Spatial regression of infant maltreatment rates on perinatal risk factors: multivariate

**Variables**	**Coefficient Estimates**		***P*-value**	**Spatial auto-correlation estimates**	**AIC**	**Pseudo-*R*^2^**
					
	**Estimates**	**Standard Error (SE)**				
Mother smoked during pregnancy	0.0221	0.003492	0.0000	0.6894*	**2573.3**	**0.564**
Three or more siblings	0.0166	0.004533	0.0002			
Unmarried mother	0.0077	0.002169	0.0004			
Maternal age <20	0.0132	0.004317	0.0023			
Medicaid beneficiary	0.0054	0.002306	0.0183			
Prenatal care less than adequate	0.0046	0.002321	0.0469			

Multicollinearity condition number (MCN) = 20.5						

*P-value < 0.0000

Table 3 presents the multivariate spatial regression results. This is the best multivariate model among several alternatives (others not shown) with the smallest AIC value. Included in the model are six risk factors: mother smoked during pregnancy, three or more siblings, maternal age <20, unmarried marital status, Medicaid beneficiary, and prenatal care less than adequate. The model has a value of 20.5 for MCN, indicating no serious problem with multicollinearity [32]. The value of pseudo-*R*^2 ^suggests the model has moderate predictive ability. All risk variables are statistically significantly with respect to the rate of infant maltreatment at least at the 0.05 level. Also, spatial autocorrelation is a significant factor with *P*-value < 0.0000.

The multivariate model provides slightly better predictivity than any of the bivariate models since it gives the smallest AIC value. However, the decrease of AIC values is only 2% compared with the best bivariate model (Model 13).

## Discussion

Several issues must be addressed to properly interpret the results: 1) ecological design, 2) spatial autocorrelation, and 3) the different types of child maltreatment.

This research used an ecological study design. Relationships found in ecological studies may suffer from two problems: the ecological fallacy and modifiable areal unit problems (MAUP) [24]. This means relationships found at the census tract level are pertinent to the current configuration of census tracts. They may not be inferred to finer levels such as block group or to individuals who lived in the areas.

We found spatial autocorrelation effects statistically significant in both bivariate and multivariate regression models. Several situations described in the Method section might be present in this study: inherent spatial dependency, missing variables, a mismatch between the unit of analysis and the unit of actual phenomena, and measurement errors. First, the dependent variable might be inherently spatially dependent. Since the observations of the response variable were not acquired through a strict sampling design but a collection of data arranged by the geographic unit, i.e., the census tract, the interdependence between observations of neighboring census tracts might be the rule rather than the exception [27]. Second, it is apparent that many variables were not included in any of the bivariate regression models. Even in the multivariate regression model, there were important variables missing. This is because child maltreatment is a social-psychological phenomenon that involves a number of risk factors, including the characteristics of the victim, the maltreater, the family, the community, and the society, as suggested by the ecological theory of child maltreatment [33,34].

The problem of mismatch between the unit of analysis and the unit of actual phenomena might also be present. This study used the census tract as a surrogate of the community. There is no compelling reason at this time to believe that child maltreatment conforms to the configuration of census tracts, except to note that tracts are designed to be demographically homogeneous. Future study using a more meaningful geographic unit may be beneficial.

The spatially correlated measurement errors in this study might result from the geocoding process. Approximately 85% of the maltreatment records were geocoded based on their address information to the accuracy of the census tract level. For the remaining 15% of the records, the county was known, but had inappropriate address information, such as P.O. boxes, or incorrect and/or incomplete addresses. For these records, a method of spatial imputation was employed in which census tracts within the county were assigned based on fertility probability, a method with an adjusted *R*^2 ^accuracy of 0.82 compared with known county fertility rates. Latitudes and longitudes were assigned randomly within selected tracts (F. Millard and G. Freymann, unpublished work, December 2001). Therefore, a record was more likely to be located in a census tract that is spatially close to the correct tract.

A final issue to address is that of the different types of child maltreatment. In this study we aggregated all types of abuse and neglect, which include neglect, physical abuse, emotional abuse, sexual abuse, and other abuse, to reduce the small number problem. It has been suggested that different types of child maltreatment involve different risk factors [35]. Therefore, it may be of benefit in future studies to separate out the types of maltreatment to determine if significant risk factors change or improve the model.

## Conclusion

Efficient allocation of resources is a priority. The method described in this paper can augment decision making regarding funding and intervention decisions.

With proper data architecture, addressing some of the shortcomings of the ecological methods used in this research can be easily overcome. For example, adjusting the spatial unit of analysis from the tract to some other form or level of precision, such as cells of varying sizes or block groups, becomes simple when data and quality standards are in place. What is less clear however, is which unit of analysis is most appropriate for the outcome of interest. In the event that "sociological meaningful scale" is unclear, analyzing the data at multiple scales may be beneficial.

Upon consideration, what at first seemed a limitation, namely the lack of unique identifiers to link individual abuse records with birth records, could actually have led to a more suitable method of analysis for the data. The method described should be used to help target interventions towards population sub-groups, as this is the purpose of public health – to assure the conditions in which people can be healthy – and does not necessarily require a person-based medical model.

One should use the ecological model to open inquiry into other social and environmental conditions present in areas with the greatest maltreatment risk. Why do some communities demonstrate the characteristics of the multivariate regression model (Table 3), i.e. high percentages of young, unmarried mothers, mothers who smoke during pregnancy, inadequate prenatal care, large families, and Medicaid recipients? What actions should be taken to alleviate these conditions/outcomes? The model lends itself to a holistic approach toward community health assessment that is based on resiliency, rather than merely presence or absence of disease or poor outcome. As stated previously, the Health Field Concept and Health Field Theory support such an approach.

The presence of spatial autocorrelation has at least two important implications: 1) an assessment model must take spatial autocorrelation into account, and 2) poor health outcomes exhibiting spatial autocorrelation indicate the need for community-level (ecological) responses. Implicit in 2) is that case-management alone will not prevent child maltreatment when larger ecological issues are not identified nor addressed.

Perhaps most importantly, is that this and similar models allow public health to take a proactive stance, and reasonably predict areas where poor outcomes are more likely to occur. U.S. states routinely collecting variables defined by NCHS for birth certificates can easily identify areas that are at high risk for infant maltreatment. This implies that public health need not be burdened by relying only on the current practice of case-management, but can put measures in place to target areas before maltreatment occurs.

## List of abbreviations

AIC – Akaike Information Criteria

DFCS – Department of Family and Children Services

DHR – Department of Human Resources

DPH – Division of Public Health

IRB – Institutional Review Board

MAUP – Modifiable Areal Unit Problem

MCN – Multicollinearity Condition Number

MLE – Maximum Likelihood Estimation

NCHS – National Center for Health Statistics

OHIP – Office of Health Information and Policy

OLS – Ordinary Least Squares

## Competing interests

The author(s) declare that they have no competing interests.

## Authors' contributions

All authors made substantial contributions to the conceptual design of the study and to the acquisition and processing of data. EJH performed the initial exploratory data analysis/cartographic visualization. YZ followed with confirmatory statistical analysis and wrote the first draft of the paper. EJH produced the presentation graphics and the final manuscript with extensive input regarding findings of relevance to public health from GRF.
